# Potential Mechanism of Action of 3′-Demethoxy-6-*O*-demethyl-isoguaiacin on Methicillin Resistant *Staphylococcus aureus*

**DOI:** 10.3390/molecules200712450

**Published:** 2015-07-08

**Authors:** Juan Manuel J. Favela-Hernández, Aldo F. Clemente-Soto, Isaías Balderas-Rentería, Elvira Garza-González, María del Rayo Camacho-Corona

**Affiliations:** 1 Facultad de Ciencias Químicas, Universidad Autónoma de Nuevo León, Av. Universidad S/N Ciudad Universitaria, San Nicolás de los Garza, Nuevo León C.P. 66451, Mexico; E-Mails: jackman610@msn.com (J.M.J.F.-H.); aldo.clemente@alumnos.uaem.mx (A.F.C.-S.); isaias.balderasrn@uanl.edu.mx (I.B.-R.); 2 Facultad de Ciencia Químicas, Universidad Juárez del Estado de Durango, Av. Artículo 123 S/N, Núcleo Universitario, Col. Filadelfia, Gómez Palacio, Durango C.P. 35015, Mexico; 3 Servicio de Gastroenterología y Departamento de Patología Clínica, Hospital Universitario, Dr. José Eleuterio González. Madero y Aguirre Pequeño, Universidad Autónoma de Nuevo León, Mitras Centro, Monterrey, Nuevo León C.P. 64460, Mexico; E-Mail: elvira_garza_gzz@yahoo.com

**Keywords:** *Larrea tridentata*, 3′-demethoxy-6-*O*-demethylisoguaiacin, methicillin-resistant *Staphylococcus aureus*, microarrays, real time PCR, mode of action

## Abstract

Bacterial infections represent one of the main threats to global public health. One of the major causative agents associated with high morbidity and mortality infections in hospitals worldwide is methicillin-resistant *Staphylococcus aureus*. Therefore, there is a need to develop new antibacterial agents to treat these infections, and natural products are a rich source of them. In previous studies, we reported that lignan 3′-demethoxy-6-*O*-demethylisoguaiacin, isolated and characterized from *Larrea tridentate*, showed the best activity towards methicillin-resistant *S. aureus*. Thus, the aim of this study was to determine the potential molecular mechanism of the antibacterial activity of 3′-demethoxy-6-*O*-demethylisoguaiacin against methicillin-resistant *S. aureus* using microarray technology. Results of microarray genome expression were validated by real-time polymerase chain reaction (RT-PCR). The genetic profile expression results showed that lignan 3′-demethoxy-6-*O*-demethylisoguaiacin had activity on cell membrane affecting proteins of the ATP-binding cassette (ABC) transport system causing bacteria death. This molecular mechanism is not present in any antibacterial commercial drug and could be a new target for the development of novel antibacterial agents.

## 1. Introduction

**Figure 1:**
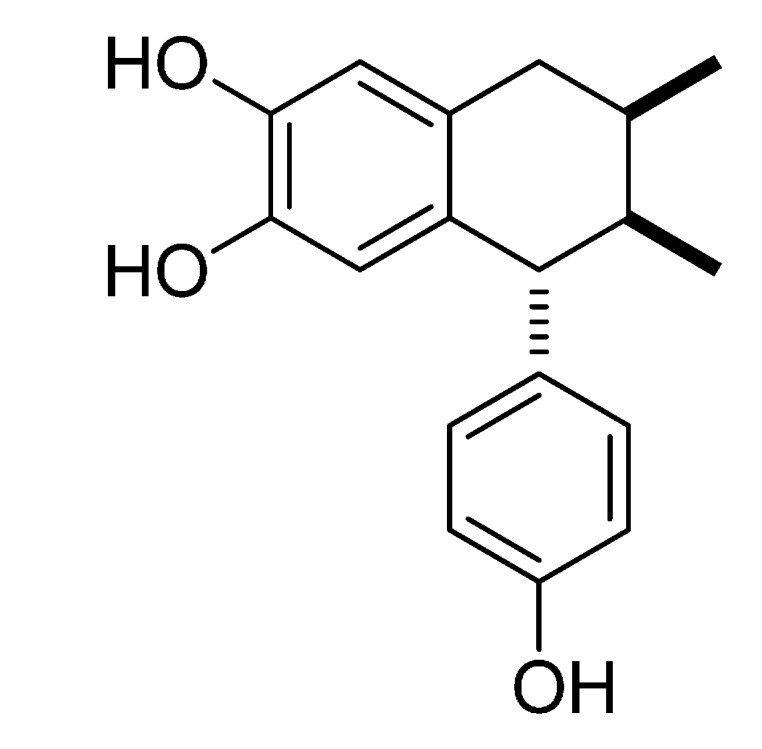
Chemical structure of 3′-demethoxy-6-*O*-demethylisoguaiacin.

Hospital-acquired *Staphylococcus aureus* infections cause some 292,000 hospitalizations and 19,000 deaths per year [1]. The worldwide emergence and dissemination of methicillin-resistant *S. aureus* (MRSA) has significantly reduced the therapeutic options and worsened clinical outcomes [2]. MRSA has spread to virtually all geographical areas for decades, arising as a major pathogen in both hospital and community settings. MRSA isolates are resistant to almost all β-lactams (except the newer anti-MRSA compounds) [3]. Therefore, there is a need to develop new antibacterial drugs with novel targets and/or mechanisms [4] and plants are a good source of them [5]. *Larrea tridentata* (Zygophyllaceae) is used in Mexico and the United States to treat bacterial infections [6]. From this plant the lignan 3′-demethoxy-6-*O*-demethylisoguaiacin (Figure 1) was isolated and characterized as the most active compound against MRSA [7]. The aim of this study was to determine the potential molecular mechanism of antibacterial activity of 3′-demethoxy-6-*O*-demethylisoguaiacin against methicillin-resistant *S. aureus* using microarray high-throughput technology and validate the results by real-time polymerase chain reaction (RT-PCR).

## 2. Results and Discussion

### 2.1. Growth Curve of MRSA with Different Concentrations of 3′-Demethoxy-6-O-demethylisoguaiacin

**Figure 2:**
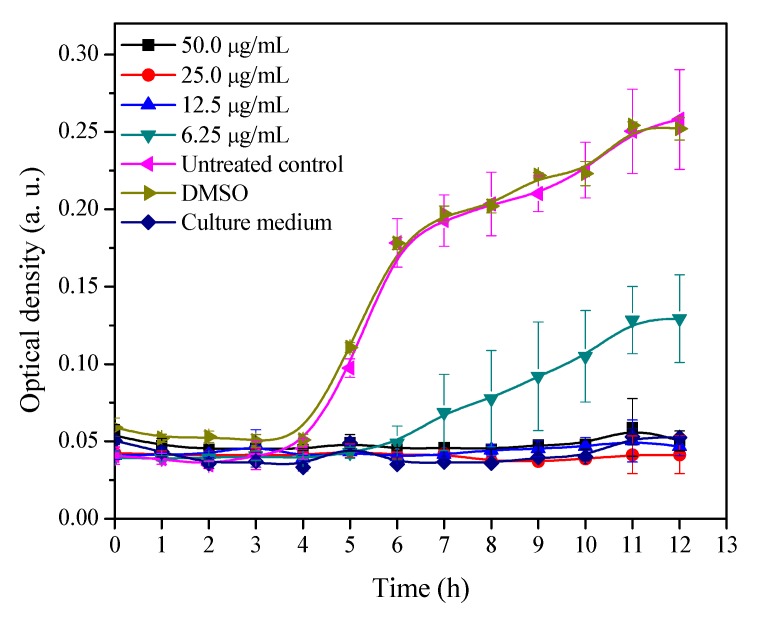
Bacterial growth-inhibitory concentration curve of 3′-demethoxy-6-*O*-demethylisoguaiacin against MRSA.

In order to determine the potential molecular mechanism of antibacterial activity of 3′-demethoxy-6-*O*-demethylisoguaiacin, a growth inhibitory curve of MRSA with different concentrations of 3′-demethoxy-6-*O*-demethylisoguaiacin was performed. Results showed that 3′-demethoxy-6-*O*-demethylisoguaiacin completely inhibited bacterial growth at concentrations of 50, 25 and 12.5 µg/mL, whereas a concentration of 6.25 µg/mL, there was growth at day 6 and growth continued until the end of the experiment but did not reach the optical density of the control (Figure 2). According to these results, the conditions (concentration and time) selected were 12.5 µg/mL and 1 h.

### 2.2. Microarray Assay and RT-PCR

The microarray results showed a group of over-expressed (122) and repressed (84) genes with a Z score of ≥+1.5 and ≤−1.5, respectively. After microarray analysis, the analyzed genes were separated into groups according to their functional category (Table 1). The microarray results may be viewed at: http://www.ncbi.nlm.nih.gov/geo/query/acc.cgi?acc=GSE70309.

**Table 1:** Functional category of over expressed and repressed genes obtained from microarrays assay.

Functional Category	Over Expressed Genes (%)	Repressed Genes (%)
Translation/structural constituent of ribosome	11.5	0
Pathogenesis	2.5	0
Oxidation-reduction process	3.3	0
Metabolic process	8.3	3.6
Unknow function, uncharacterized protein	14.3	37.8
Transcription	3.3	0
Translation	3.3	1.2
Catalytic activity	2.5	0
Biosynthetic process	7.5	8.4
Catabolic process	2	0
Amino acid transport	2.5	0
Transport protein	0	4.8
Glycolisis	0	2.4
DNA repair	0	2.4
Proteolysis	0	2.4
Mechanism of defense	0	4.8
Individual genes with different biological function	39	32.2

Some over-expressed genes are related to three metabolic pathways: pyruvate (SAR0217, SAR0824, and SAR0169), propanoate (SAR0217, SAR0169) and butanoate (SAR0217, SAR0169). These genes are related to catalytic activity, and in biological systems, they are essential for normal life processes. It is important to point out that the genes mentioned lower the energy required to begin a reaction, and decrease the reaction rate of enzymatic pathways at which cells perform essential chemical reactions [8].

Furthermore, catalysis is controlled by regulating enzymes, some of which are involved in enzyme structure changes, while others operate by altering the cell environment for catalytic activity. The molecular functions and the metabolic process involved in this biological process depend on the catalytic activity of enzymes that mediate most cell functions such as metabolism, which increases the rate of virtually all chemical reactions in cells in order to sustain life. Without enzymatic catalysis, most biochemical reactions would be so slow that they would not occur. Cells contain thousands of different enzymes, and their catalytic activities determine many chemical reactions that take place in the cell [9]. We propose that over-expression of the previously mentioned genes related with regard to catalytic activity affect main bacterial processes. Table 1 showed that the SAR1963 genes “Regulation of DNA repair” and SAR0785 “DNA replication” were also affected in MRSA. SAR1963 is part of a collection of processes by which a cell identifies and corrects damage to the DNA molecules that encode its genome, but genetic over-expression generates genetic diversity. This cause the losing of genomic stabilility as well as the lack of coordination and detection to a response to the damage, without effecting measures to prevent cell death [10,11]. The SAR0785 gene is directly related to the cellular metabolic process in which a cell duplicates one or more DNA molecules. Genetic over-expression affects the origin of replication particularly the genome sequence at which replication is initiated [12,13].

Analysis of repressed genes with the DAVID database resulted in some ATP-binding cassette (ABC) transport system proteins. This system is universally distributed among living organisms and functions in many different aspects of bacterial physiology [14]. This system is characterized by ABC protein transporters located in the cell membrane. Molecules are transported inside and outside the cell and this facilitate drugs release and elimination of toxic substances. In this way the therapeutic effects of antibacterial agents are avoided [15,16,17]. Zechini and Versace reported that some proteins of the ABC system are associated to drug-resistance [18]. Resistance of *S. aureus* RN4220 to vancomycin is due to the transport protein “msrA”; *M. tuberculosis* is resistant to tetracycline due to the protein “DrrAB”; *E. coli* is resistant to erythromycin due to the protein “MAcAB-TolC”. The ABC transport system of *S. aureus* is formed by 91 genes that express different proteins of this system. Of these 91 genes, we found six that were repressed (SAR0144, SAR1073, SAR1928, SAR0306, SAR0618, and SAR2267) after treatment with 3′-demethoxy-6-*O*-demethylisoguaiacin. With this we suggest that the above phytochemical compound could exert its effect on this membrane transport system.

The genes SAR0144, SAR1073, SAR1928 have a molecular function as ABC-type transporter ATP-binding protein, SAR0306 is a lipoprotein that is a releasing system ATP-binding protein, SAR0618 is a transport lipoprotein related to the union-substrate system and SAR2267 belongs to a subfamily of transport proteins, FecCD, and is related with ABC transporter permease system. 3′-demethoxy-6-*O*-demethylisoguaiacin repressed these MRSA proteins, and the bacteria could not expel the phytochemical, therefore this compound demonstrated its antibacterial activity.

In order to validate the expression level of six down-expressed genes (Table 2) corresponding to the ABC transport system, a RT-PCR assay was performed. In all six selected genes, repressed levels of mRNA as a result of exposure to the phytochemical compound were confirmed. This provides support to the microarray results and the above proposals.

**Table 2:** Real-Time PCR of six repressed genes involved in ABC transport system.

Gen ID	Description	Forward 5′→3′ Reverse 5′→3′	Zscore	RQ	Tm Dissociation
SAR0144	ABC transporter ATP-binding protein	GCACTAGAACGGGTCAACA TGGGTCTAATGAAGCAACTGG	−1.507	−5.922 ± 0.186	78.12
SAR1073	ABC transporter ATP-binding protein	ATGTTGTTTAGAGGGGTCCAC CCAACTTCGCTGCCTACT	−1.834	−5.780 ± 0.320	74.85
SAR1928	ABC transporter ATP-binding protein	TGAAGTCGTTGCATTTGGAG TCGCTTGGTTACGCATGT	−1.683	−2.378 ± 0.042	76.95
SAR0306	ABC transporter ATP-binding protein	CGATTGGGTAGGAGGTGTA CCAGAAGGTCCAACTAATGC	−1.782	−2.039 ±0.307	74.91
SAR0618	Transport system lipoprotein	GAACGCAGTTGGATGTAACC CATACCACAGCCACTCAGAA	−1.917	−1.392 ±0.061	77.04
SAR2267	FecCD transport family protein	GCGCCTTTATTGGTGGATTA TGAACCAACAAGCCAAAACA	−1.955	−4.205 ±0.028	76.56
SARr016	16S ribosomal RNA (Reference gen)	CAGCATGCTACGGTGAATAC GTTACGACTTCACCCCAATC	-	1	76.75

## 3. Experimental Section

### 3.1. Isolation, Characterization and Evaluation of 3′-Demethoxy-6-O-demethylisoguaiacin

Dried and grounded leaves (500 g) of *L. tridentata* were extracted by maceration, first with hexane and then with CHCl_3_. Both extracts were separately distilled *in vacuo* until dryness. A total of 89 g of chloroform extract was obtained. A portion of this extract (80 g) was subjected to silica gel column chromatography (CC) and eluted with gradient of CHCl_3_/MeOH yielding 13 fractions. Fraction 10 (18 g) was chromatographed three times on silica gel CC, each column was eluted with a gradient of CHCl_3_/MeOH. From the sub-fraction 42–73 of the third column precipitated a brown solid which was purified by recrystallization with CHCl_3_/MeOH (98:2) yielding a beige solid (142.4 mg) [7]. This compound was characterized as 3′-demethoxy-6-*O*-demethylisoguaiacin by comparison of its spectral data with literature [19].

3′-Demethoxy-6-*O*-demethylisoguaiacin was evaluated against MRSA showing a MIC value of 12.5 µg/mL, which was the same as the positive control levofloxacin [7]. The cytotoxicity of the above natural product was determined using the WST-1 methodology [20]. Results showed that 3′-demethoxy-6-*O*-demethylisoguaiacin has an IC_50_ value of 31.10 µg/mL whereas the positive standard vincristine showed an IC_50_ value of 0.48 μg/mL against Chang liver cells (ATCC CCL-13) [21].

### 3.2. Growth Inhibitory Curve of MRSA Exposed to Different Concentrations of 3′-Demethoxy-6-O-demethylisoguaiacin

A stock solution was made by dissolving 1 mg of natural product in dimethylsulphoxide (DMSO) to reach a concentration of 20 mg/mL. A working solution was made with 3 μL of stock solution and 297 µL of Mueller-Hinton broth to give a concentration of 200 μg/mL. Then 100 µL of Mueller-Hinton broth was added to each well of a sterile 96 well microplate. Then, 100 μL of the working solution was added to row A. From this mixture 100 µL was taken with a multichannel pipet and transferred to row B to perform a 1:2 dilution. Then, further 1:2 dilutions were made until row D, finally the last 100 μL was discarded. Further, 100 µL of MRSA ATCC BAA-44 in Mueller-Hinton (105) was added to the wells with and without sample. The compound was tested in the concentrations of 50, 25, 12.5 and 6.25 µg/mL of 3′-demethoxy-6-*O*-demethylisoguaiacin. DMSO in each well did not exceed 0.05%, and this concentration does not affect bacterial growth. Each concentration was evaluated in triplicate. Two untreated controls were made, one with only bacteria and the other with bacteria and DMSO (≤0.083%). The plate was covered with a lid and incubated a 37 °C. Bacterial growth was determined by measuring the optical density at 620 nm at regular intervals of one hour until completing 12 h using a 96-well microtiter plate reader. The bacterial growth curve was performed in order to determinate exposure time and the concentration of 3′-demethoxy-6-*O*-demethylisoguaiacin needed to cause stress on MRSA. The experiment was done three times on different days.

### 3.3. RNA Isolation and Synthesis of Labeled cDNA

Two Erlenmeyer flasks with 100 mL of Mueller-Hinton broth each were inoculated with MRSA and the turbidity was adjusted at 0.5 MacFarland’s standard (1.5 × 10^8^ CFU). Both flasks were incubated for 4 h to achieve logarithmic phase (optical density 0.4 at 600 nm), then drug treatment was conducted by adding 3′-demethoxy-6-*O*-demethylisoguaiacin to one of the cultures to achieve a final concentration of 12.5 μg/mL. Untreated control bacteria was grown under identical conditions to treated bacteria, with the exception that no drug was added, only dimethyl sulfoxide (DMSO) which did not exceed 0.05% *v*/*v*. Both flasks were incubated 1 h during drug treatment. After incubation time the bacteria was harvested separately by centrifugation at 3500 rpm for 15 min. The bacterial pellets obtained were stored at −70 °C for RNA isolation. Total RNA of each bacterial pellet was isolated and purified with the RiboPure-Bacteria AM1925 kit (Ambion, Carlsbad, CA, USA) according to the manufacturer’s instructions, including a DNAse digestion step. Quantity and quality of RNA obtained were assessed by performing agarose gel electrophoresis and analyzing this in a biophotometer and Molecular Imager^®^ Gel Doc™ XR^+^ System equipped with Image Lab™ software, respectively (BioRad, Hercules, CA, USA). To obtain the mRNA from each total RNA (10 µg), the MICROBExpress AM1905 (Ambion) kit was used according to the manufacturer’s protocol. The Labeled cDNA of each condition was obtained from mRNA using Amino Allyl cDNA Labeling AM 1705 (Ambion) kit and Amersham CyDye Post-Labeling Reactive Dye Packs (GE Healthcare Bio-Sciences AB, Piscataway, NJ, USA) according to the manufacturer’s instructions.

### 3.4. Microarray and Data Analysis

Microarray hybridization was performed using the procedure described in the protocol by MYcroarray Company (Ann Arbor, MI, USA). A whole genome of *S. aureus* MRSA252 (CAT-30K) consisting of 1 piece of sequence (BX571856 GeneBank) representing a total of 2744 transcripts was used. Dynamic hybridization was performed in a hybridization Chamber G2534A (Agilent, Santa Clara, CA, USA) and covered with G2534-60002 gasket slides (Agilent). Fluorophore incorporation efficiency was analyzed measuring absorbance at 555 nm for Cy3 and 655 nm for Cy5 using a Gene Pix 4000B scanner with Acuity 4.0 software (Molecular devices, Sunnyvale, CA, USA); scans were saved as TIFF images and quantified. Data were then normalized and analyzed with genArise software (version 2.2.0, Computing Unit, Cellular Physiology Institute, UNAM, Mexico City, Mexico). Genes with a Z score ± 1.5 were considered with altered expression. The list of genes with altered expression was analyzed with the following bioinformatic tools databases: Data base for Annotation Visualization and integrated Discovery (DAVID) [22], Kyoto Encyclopedia of Genes and Genomes (KEGG) [23], and GenBank, GenDB [24]. In order to perform this analysis, the list of genes of interest was inserted into these tools and databases. A group of genes with function was obtained and organized in Table 1.

### 3.5. Real Time-PCR Assay

Samples of both treated and untreated mRNA isolated from MRSA were employed in RT-PCR. cDNA was obtained using SuperScript^®^ VILO™ cDNA Synthesis Kit (Invitrogen 11754050, Life Technologies, Carlsbad, CA, USA). RT-PCR was performed using SYBR^®^ GreenER™ qPCR SuperMix Universal (Invitrogen 11780200, Life Technologies). The relative quantitation method employed was the comparative C_T_ in StepOne™ Real-Time PCR system (Applied Biosystems, Foster City, CA, USA). The specific primers used in the experiment were designed in primer3 version 4. In order to assure specific amplification, melting curves were performed for all interest genes and the samples were run in triplicate in each reaction; 16S ribosomal RNA (SARr016) was used as housekeeping gene.

## 4. Conclusions

We propose a potential molecular mechanism for 3′-demethoxy-6-*O*-demethylisoguaiacin whereby it acts on the cellular membrane where the phytochemical represses some proteins of the ABC transport system of MRSA. Thus the bacteria could not release the phytochemical and this caused its antibacterial activity. It is important to point out that this molecular mechanism is not presented by any commercial drug and could be a new target for the development of novel antibacterial agents.
